# Complete chloroplast genome of *Lonicera ligustrina* Wall. (Caprifoliaceae) and its phylogenetic implications

**DOI:** 10.1080/23802359.2023.2239386

**Published:** 2023-08-25

**Authors:** Li-Li Liu, Jian-Hui Li, Zhong-Shuai Sun

**Affiliations:** a College of Chemistry and Materials Engineering, Quzhou University, Quzhou, China; b Quzhou Academy of Agricultural and Forestry Sciences, Quzhou, China; c Zhejiang Provincial Key Laboratory of Plant Evolutionary Ecology and Conservation, Taizhou University, Taizhou, China

**Keywords:** *Lonicera ligustrina*, *lonicera*, chloroplast genome, phylogenomics

## Abstract

*Lonicera ligustrina* is a folk medicinal herb in China and India with highly potential medicinal value. Here, we reported the complete chloroplast (cp) genome of *L. ligustrina* (GenBank accession number: ON968694). The cp genome was 155,330 bp long, with a large single-copy region (LSC) of 88,855 bp and a small single-copy region (SSC) of 18,647 bp separated by a pair of inverted repeats (IRs) of 23,914 bp. We also reconstructed the phylogeny of *Lonicera* using maximum likelihood (ML) method, including our data and previously reported cp genomes of related taxa. The current study indicated that *L. ligustrina* is sister with the *Nintooa* clade of subgen. *Lonicera*.

## 1. Introduction

The genus *Lonicera* L., commonly known as honeysuckles, is the largest genus of Caprifoliaceae (Ren et al. 2008; Jacobs et al. 2009). Historically, it has received extensive taxonomic evaluation and phylogenetic inference, including studies based on complete chloroplast genome (Smith and Donoghue 2010; Fan et al. 2018; Wang et al. 2020), but so far, its phylogenetic relationships among sections, subsections, and species are still obscure (Theis et al. 2008; Nakaji et al. 2015; Wang et al. 2020). *Lonicera ligustrina* Wall.1824, is an evergreen, semi-evergreen, or deciduous shrub belonging to the section *Isika* of Caprifoliaceae (Hsu and Wang 1988). It can be classified into 3 varieties: *L. ligustrina* var. *ligustrina*, *L. ligustrina* var. *pileata* (Oliver) Franchet, *L. ligustrina* var. *yunnanensis* Franchet (Yang and Landrein 2011). The species varied greatly in morphology, and more research work is still needed to clarify its infraspecific classification (Zeng et al. 2021). At the same time, *L. ligustrina*, commonly used in folk herbs in China and India also has certain medicinal value (Yashodha et al. 2019; Murthy et al. 2022). Therefore, we assembled and characterized the complete chloroplast genome of *L. ligustrina*, which is expected to lay a solid foundation for the medical applications and future phylogenetic investigations of *Lonicera*.

## 2. Materials and methods

### 2.1. Plant materials and DNA extraction

**Figure 1.**
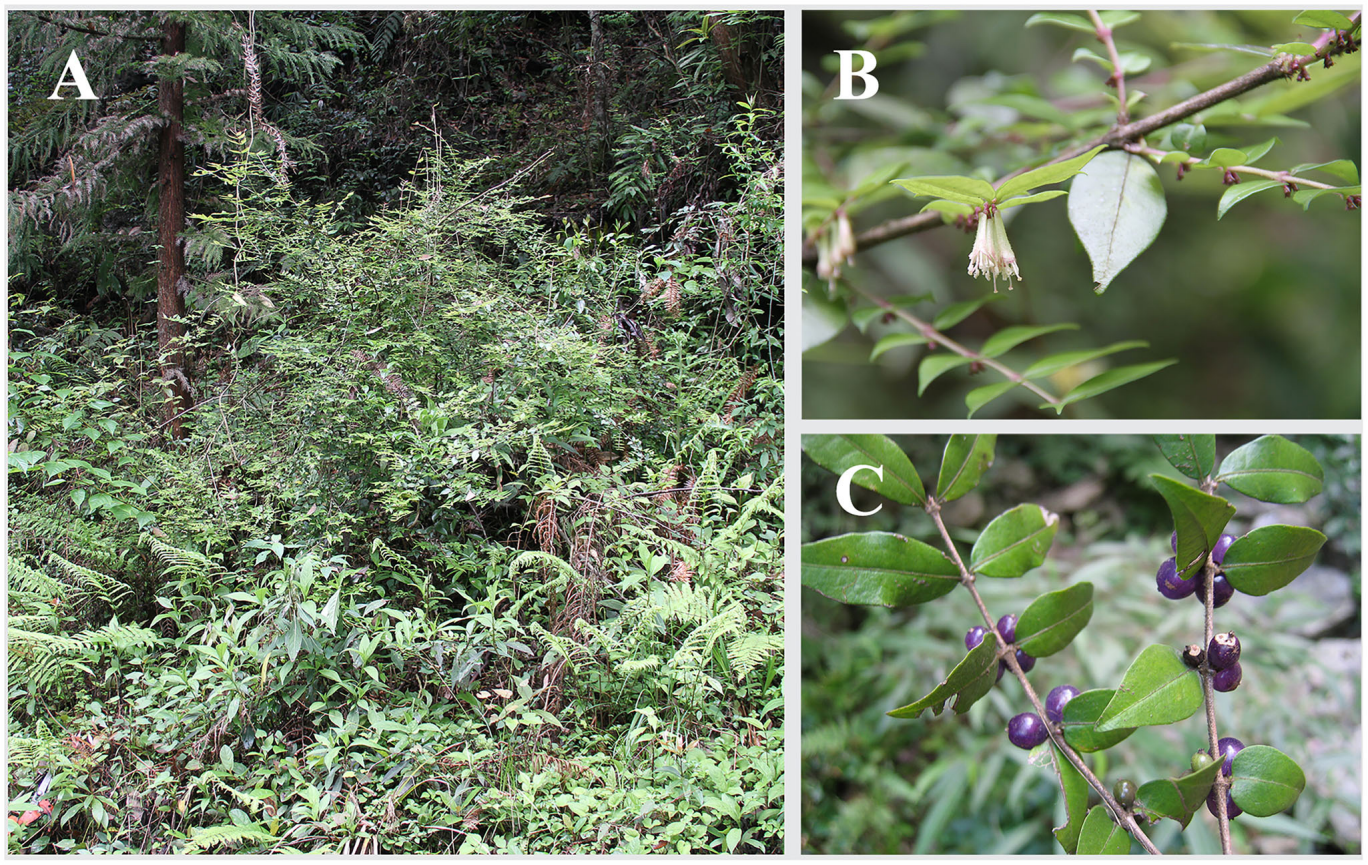
Pictures of *L. ligustrina* taken at Baiguoba, Enshi, Hubei province, China. A. natural habitat; B. flowering branches; C. fruit branches and leaves.

Fresh leaves of *L. ligustrina* (Figure 1) were collected from Baiguoba, Enshi, Hubei province, China (30.2681 N, 109.4008E). A voucher specimen was deposited at Herbarium of Taizhou University (https://www.tzc.edu.cn/; collector: Zhong-Shuai Sun, sun2143998@163.com) under the voucher number ES548. Genomic DNA was extracted as reported in Chen et al. (2022).

### 2.2. Plastome sequencing, assembly, and annotation

The next-generation sequencing was performed with an Illumina NovaSeq platform (Illumina, San Diego, CA). The chloroplast genome was assembled *via* NOVOPlasty 2.6.3 (Dierckxsens et al. 2017), using the *L. japonica* (NC026839, He et al. 2017) as the initial reference genome. The assembled cp genome was annotated using the online software GeSeq v.1.59 (Tillich et al. 2017). Geneious R11 (Biomatters Ltd., Auckland, New Zealand) was used for inspecting the cp genome structure. The circular gene map of the chloroplast genome was drawn by d by CPGView software (Liu et al. 2023, http://www.1kmpg.cn/cpgview/).

### 2.3. Phylogenetic analysis

Species phylogeny of *Lonicera* was evaluated based on protein coding regions (CDS) extracted from 27 complete chloroplast genomes from Caprifoliaceae. *Heptacodium miconioides* (NC042739, Wang et al. 2019) was used as outgroup. We reconstructed a phylogeny employing the GTR + G model and 1000 bootstrap replicates under the maximum likelihood (ML) inference in RAxML-HPC v.8.2.10 on the CIPRES cluster (Miller et al. 2010).

## 3. Results

### 3.1. Genome organization and compositions

**Figure 2.**
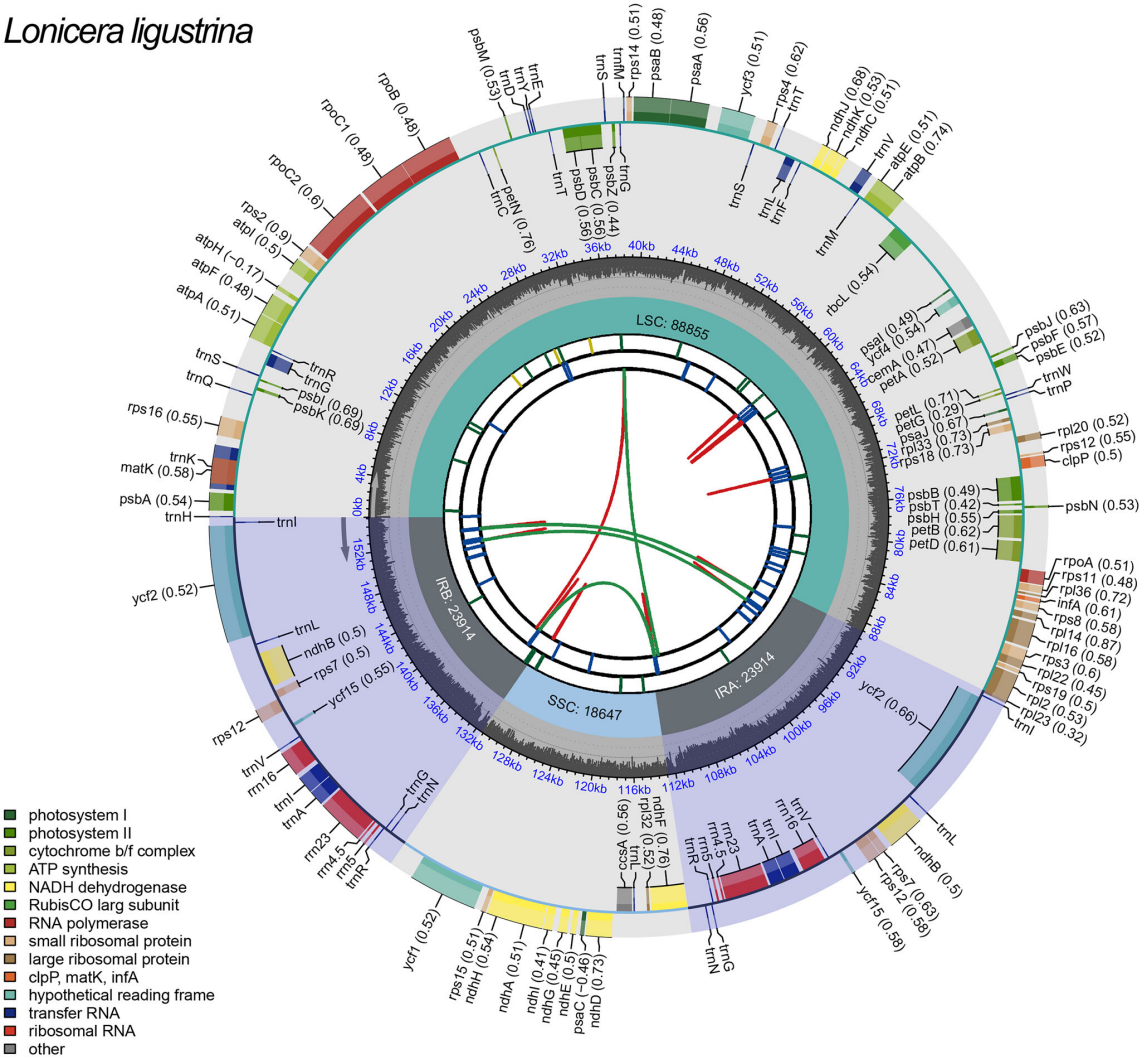
Schematic map of *L. ligustrina* chloroplast genome constructed by CPGview (http://www.1kmpg.cn/cpgview/). The functional classification is shown at the bottom left.

The complete chloroplast genome of *L. ligustrina* is 155,330 bp in length. It has a typical quadripartite structure with a large single-copy (LSC) region of 88,855 bp, a small single-copy (SSC) region of 18,647 bp, and a pair of inverted repeats (IRs) of 23,914 bp (Figure 2). The GC contents of the total length, LSC, SSC, and IR regions were 38.5%, 36.9%, 33.4% and 43.3%, respectively.

The genome contains a total of 129 functional genes, including 84 protein-coding genes (PCGs), 37 tRNA genes and 8 rRNA genes. Most genes occurred in a single copy, while 5 PCGs (ycf2, ndhB, rps7, rps12 and ycf15), 7 tRNA genes (trnI-CAU, trnL-CAA, trnV-GAC, trnI-GAU, trnA-UGC, trnR-ACG and trnN-GUU), and 4 rRNA genes (rrn4.5, rrn5, rrn16, rrn23) in IR regions are duplicated. Of all these genes, 15 genes (rps16, atpF, rpoC1, petB, petD, rpl16, rpl2, ndhB, ndhA, trnK-UUU, trnG-UCC, trnL-UAA, trnV-UAC, trnI-GAU, and trnA-UGC) had one intron and 3 genes (rps12, rps18 and ycf3) had two introns.

### 3.2. Phylogenetic analysis

**Figure 3.**
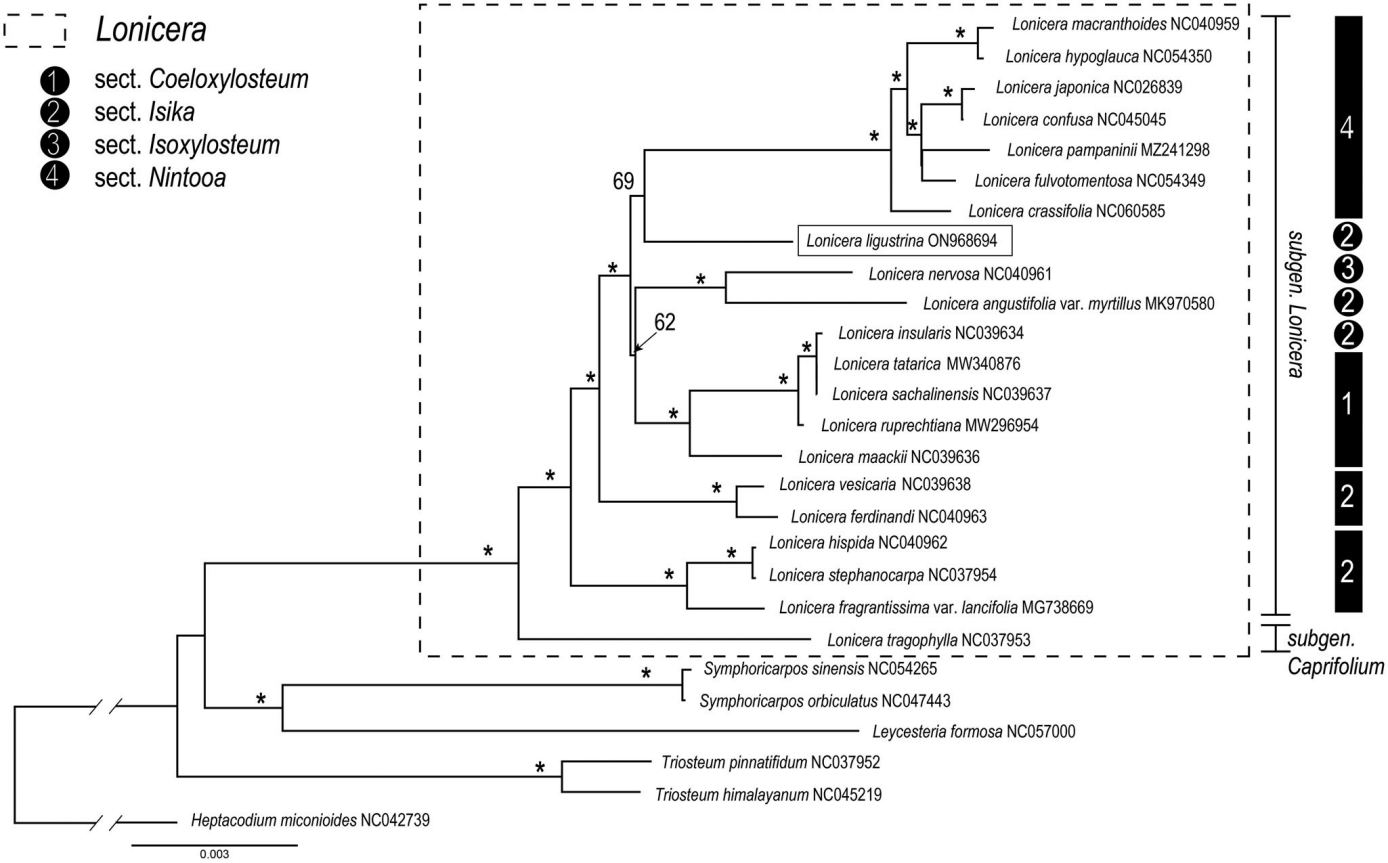
Maximum likelihood (ML) tree reconstruction of 27 taxa from Caprifoliaceae based on 84 shared CDS in the chloroplast genomes. Relative branch lengths are indicated. Support values above the branches are ML bootstrap support; ‘*’ indicates 100% support values. The following sequences were used: NC042739 (Wang et al. 2019), NC045219 (Liu et al. 2019), NC057000 (Zhang 2020), NC047443 (Shen et al. 2019), NC054265 (Zhang et al. 2020), NC037952 (Fan et al. 2018), NC037953 (Fan et al. 2018), MG738669 (Fan et al. 2018), NC037954 (Fan et al. 2018), NC040961 (Liu et al. 2018), NC040962 (Liu et al. 2018), NC040963 (Liu et al. 2018), NC039636 (Jia et al. 2020), NC039637 (Jia et al. 2020), NC039638 (Jia et al. 2020), NC039634 (Jia et al. 2020), MW296954 (Gu et al. 2022), MW340876 (Yuan et al. 2021), MK970580 (Wu et al. 2021), NC060585 (Chen et al. 2022), NC054349 (Yu et al. 2021), NC054350 (Gu et al. 2021), MZ241298 (Jiang et al. 2021), NC045045 (Wang et al. 2019), NC026839 (He et al. 2017); NC040959 (Hu et al. 2018).

A robust phylogeny of *Lonicera* was obtained based on the CDS data, most nodes in the ML tree were highly supported, and the genus was resolved as a monophyletic clade consisting two well supported clades which consistent with the previous research which divided *Lonicera* into two subgenera: subgen. *Caprifolium* and subgen. *Lonicera* (Figure 3). The subgen. *Lonicera* clade consisted of species from 4 sections (sect. *Coeloxylosteum*, sect. *Isika*, sect. *Isocylosteum*, sect. *Nintooa*), but in our phylogenetic tree, only the species from sect. *Nintooa* were clustered into a monophyletic clade. According to the phylogenetic tree, the sister relationship between *L. ligustrina* and *L. ligustrina* clade was observed.

## 4. Discussion

The current study presented the complete chloroplast genome of *L. ligustrina* and a highly resolved phylogenies of *Lonicera* based on 27 complete chloroplast genome from Caprifoliaceae. Our result supports the classification of the two subgenera, subgen. *Lonicera* and subgen. *Caprifolium*, in *Lonicera* proposed by Rehder (1903, 1913) and Hsu and Wang (1988) and coincides with previous molecular phylogenetic studies (Theis et al. 2008; Nakaji et al. 2015; Wu et al. 2021). Among the four sections of subgen. *Lonicera*, sect. *Nintooa* was supported as monophyly, and *L. ligustrina* is sister with the *Nintooa* clade with strong bootstrap support (Figure 3). We expect that the cp genome of *L. ligustrina* will be a valuable resource for future studies on molecular identification and the better understanding of phylogeny in *Lonicera* and Caprifoliaceae.

## Supplementary Material

Supplemental Material

Supplemental Material

Supplemental Material

## Data Availability

The genome sequence data that support the findings of this study are openly available in GenBank of NCBI at (https://www.ncbi.nlm.nih.gov/) under the accession no. ON968694. The associated BioProject, SRA, and Bio-Sample numbers are PRJNA894792, SRR22061799 and SAMN31469260 respectively.
