# Supplementary figures and images for: Complete chloroplast genome of Lonicera ligustrina Wall. (Caprifoliaceae) and its phylogenetic implications
Source: Mitochondrial DNA B Resour. 2023 Aug 25;8(8):903–7. doi: 10.1080/23802359.2023.2239386 (PMC10461517; doi:10.1080/23802359.2023.2239386)

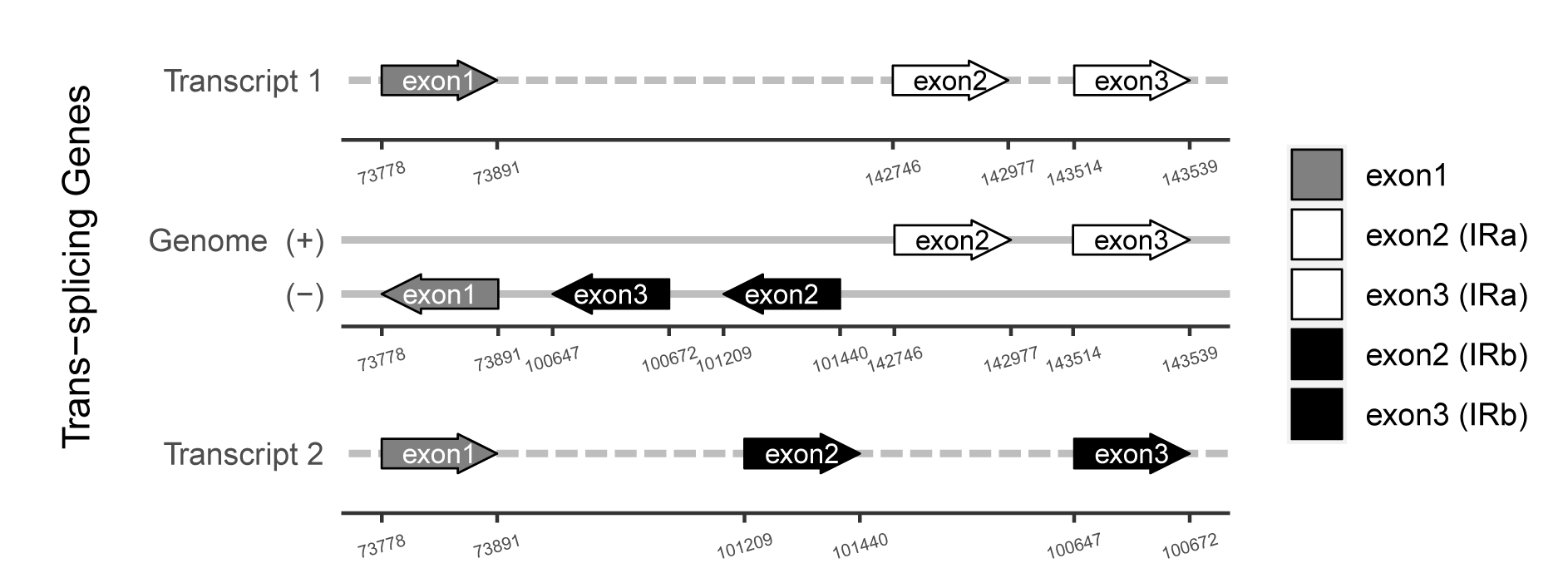

Supplement: Supplemental Material [file TMDN_A_2239386_SM5314.tif]

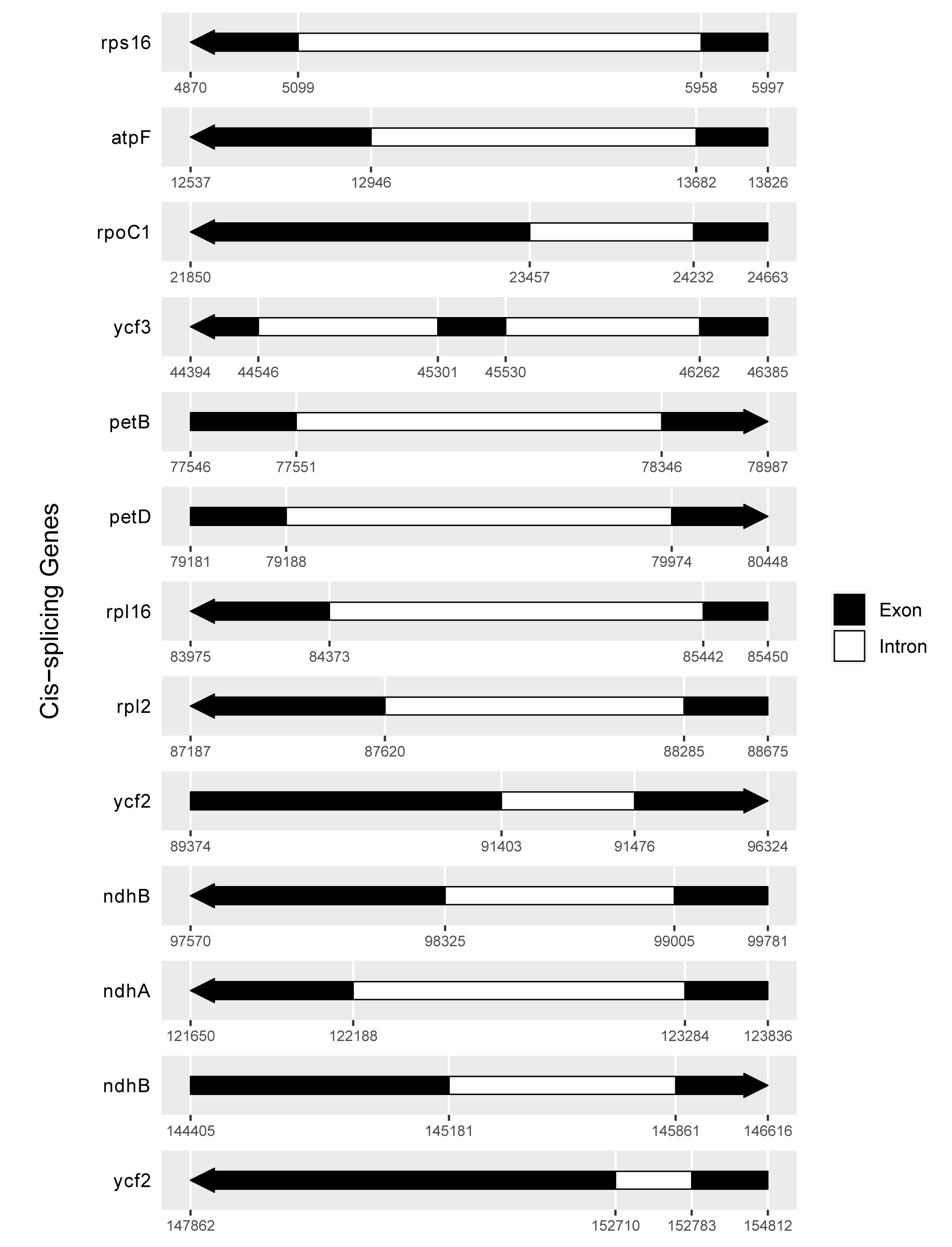

Supplement: Supplemental Material [file TMDN_A_2239386_SM5311.tif]

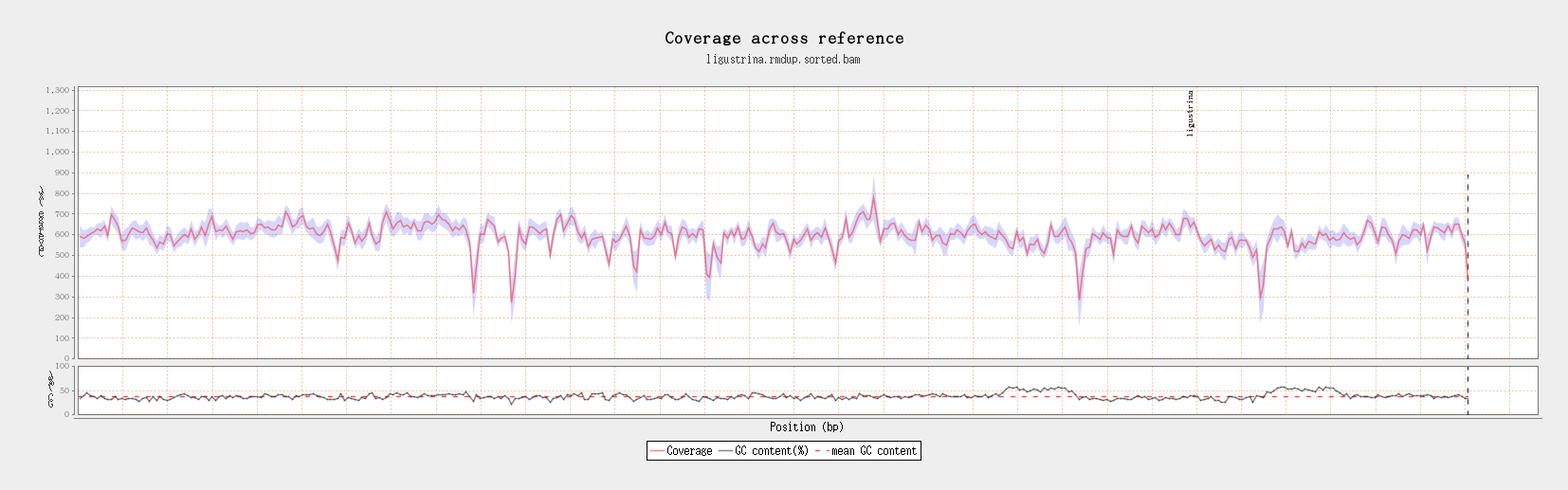

Supplement: Supplemental Material [file TMDN_A_2239386_SM5030.tif]
